# Biomechanical comparison of a new minimally invasive technique versus conventional plating for the treatment of open book symphyseal injuries in osteoporotic human pelvises

**DOI:** 10.1002/jeo2.70636

**Published:** 2026-01-19

**Authors:** Tobias Fritz, Jeremy Briem, Marcel Orth, Laura Mettelsiefen, Jonas Stroeder, Alexa J. Fischer, Emmanouil Liodakis, Tim Pohlemann, Antonius Pizanis, David B. Osche

**Affiliations:** ^1^ Department of Trauma, Hand and Reconstructive Surgery Saarland University Medical Center Homburg Germany; ^2^ Department of Trauma and Reconstructive Surgery BG Hospital Tübingen Tübingen Germany; ^3^ Department of Diagnostic and Interventional Radiology Saarland University Medical Center Homburg Germany; ^4^ Department of Radiology and Nuclear Medicine University Medical Center Schleswig‐Holstein Campus Lübeck Lübeck Germany

**Keywords:** biomechanical analysis, internal fixator, open book injuries, pelvic ring fractures, pubic symphysis

## Abstract

**Purpose:**

Traumatic open book injuries of the pubic symphysis require stable fixation while minimizing surgical morbidity. Traditional symphyseal plating is associated with complications such as implant failure and infections. This study aims to evaluate the biomechanical performance of a novel minimally invasive technique using an internal fixator (IF) compared to conventional plating (symphyseal locking dynamic compression plate [SLDCP]) in human cadaveric pelvises.

**Methods:**

Ten human cadaveric pelvises were assigned to two groups (*n* = 5 each). After anatomical reduction, either SLDCP or IF stabilization was applied. Intrasymphyseal compression forces, contact area and interfragmentary motion were assessed using pressure‐sensitive sensor films and an optical tracking system under incrementally applied axial loads up to 600 N. Bone density and symphyseal dimensions were measured and were comparable across both groups.

**Results:**

At key loading stages (400 and 600 N), the IF group demonstrated significantly higher intrasymphyseal compression forces (IF 61.78 ± 31.98 N vs. SLDCP 15.98 ± 8.2 N at 400 N and IF 42.82 ± 25.2 N vs. SLDCP 9.42 ± 5.81 N at 600 N) and larger contact areas (IF 453 ± 137.6 mm^2^ vs. SLDCP 216 ± 135 mm^2^ at 400 N and IF 337.6 ± 168.45 mm^2^ vs. SLDCP 154 ± 59.73 mm^2^ at 600 N), with more homogeneous segmental force and area distributions. Higher compression forces, particularly in the caudal symphyseal region (IF 23.68 ± 18.69 N vs. SLDCP 13.78 ± 10.1 N), were achieved. Three‐dimensional analysis showed reduced fragment displacement in the IF group under load.

**Conclusion:**

The proposed technique using an internal fixator provided superior biomechanical stability compared to standard plating, offering a promising minimally invasive alternative for managing open book injuries. The technique facilitates uniform force transmission and improved contact conditions, which may enhance healing and reduce complications.

**Level of Evidence:**

N/A.

**Abbreviations**
ANOVA: analysis of variance
CT: computed tomography
DICOM: Digital imaging and Communications in Medicine
HU: Hounsfield units
IF: internal fixator
SLDCP: symphyseal locking dynamic compression plate

## INTRODUCTION

Pelvic ring injuries account for up to 3% of all skeletal fractures [15], with a rising global incidence [1, 2, 18, 20]. Acute traumatic symphyseal instabilities are rare but can commonly be seen in polytraumatized patients, predominantly affecting male patients. The typical injury mechanism involves a direct anteroposterior blunt force to the symphysis, commonly seen in motorcycle accidents (pelvis impacting the fuel tank), horseback riding [19] (pelvis against the horse's neck), and—as some reports suggest—waterslide incidents [29] (due to water surface impact). These injuries are typically classified as ‘open book’ lesions (AO/Tile Type B1.1). Other mechanisms resulting in transsymphyseal injuries often involve concurrent posterior pelvic ring disruptions and are classified as AO/Tile Type B or C injuries. In contrast, pelvic injuries involving the symphysis are rare in the geriatric population, where low‐energy fragility fractures predominate.

The current gold standard for treating these injuries is symphyseal plate osteosynthesis, often combined with posterior ring stabilization as needed. While generally effective, this approach is associated with complications such as implant failure, soft tissue infections [6, 10, 21, 23] and mechanical and biological drawbacks associated with locking plate fixation [4]. In response, minimally invasive techniques have been introduced over the past decade. However, their widespread adoption has been limited due to steep learning curves, extended radiation exposure and inferior biomechanical stability.

To address these limitations, we developed a minimally invasive technique that combines a streamlined approach with well‐established implants. In previous studies using composite pelvises, our group demonstrated the biomechanical superiority of this method, and its clinical feasibility has also been confirmed. Given that minimally invasive approaches have gained more importance over the last decade by minimizing soft tissue trauma and lowering the risk of wound infections, there is a need for improvement in the treatment of symphyseal injuries [26].

The aim of the present study was to validate these findings using human cadaveric specimens. Although composite pelvises provide standardized dimensions and properties, especially concerning the symphysis [13], the lack of soft tissue with its unclear contribution to biomechanical properties remains a problem [22]. Also, studies have shown that the stiffness of a composite model is much higher compared to that of a human cadaveric specimen [13]. Furthermore, it has also been shown that, considering implant performance for stabilization of acetabular fractures, there is a relation between the implant's stiffness and the stiffness of the bone [8]. Taking these facts into account, validation of previous results taken from experiments with composite bone models using cadaveric specimens remains an important concept. It allows for further assessment of the biomechanical advantages of this novel technique.

In this study, we aimed to evaluate whether internal fixation of the symphysis using a minimally invasive internal fixator (IF) provides superior biomechanical stability compared to conventional symphyseal plating in human cadaveric pelvises. We hypothesized that internal fixation provides superior biomechanical stability compared to conventional plating, as reflected by higher compressive forces and more uniform contact distribution at the symphysis under cyclic loading.

## MATERIALS AND METHODS

### Specimens

For the biomechanical studies, we analyzed 10 fresh‐frozen human anatomical pelvises. Surrounding soft tissue was meticulously removed, and the hip joints were exarticulated. Special attention was paid to preserving the ligamentous structures stabilizing the sacroiliac joints (e.g., sacrotuberous and sacrospinous ligaments). The ligaments stabilizing the pubic symphysis, however, were completely removed in order to simulate an unstable transpubic injury. The pelvises were subsequently stored at −20°C. Prior to each testing series, one pelvis was thawed at room temperature for 24 h.

To ensure homogeneous group allocation, all specimens underwent computed tomography (CT) imaging. This served both to exclude the presence of implants and to assess osteoporotic bone (Table 1). Scans were acquired using a clinical 128‐slice spiral CT scanner (Definition Edge; Siemens) at 120 kV and 300 mAs. DICOM images were transferred to the Horos software (Horos Project) for further analysis of bone morphology. Bone density was quantified according to a previously published protocol by Zou et al. based on Hounsfield units (HU) measured in the centre of the S1 vertebral body in both axial and sagittal planes [31], defining thresholds for diagnosing osteoporosis of 202 HU in axial planes and 185 HU in sagittal planes. Additionally, the symphyseal surface area of each pelvis was calculated from the CT data.

**Table 1:** Demographic data of body donors.

	Bone mineral density (HU)	Age (years)	Sex
Internal fixator	214 (axial)	77.7	3 male, 2 female
	146.7 (sagittal)		
SLDCP	190 (axial)	79.5	3 male, 2 female
	149.5 (sagittal)		

*Note*: Allocation of body donors according to bone mineral density (measured in axial and sagittal CT planes, mean values), age (mean) and sex.

Abbreviations: CT, computed tomography; HU, Hounsfield units; SLDCP, symphyseal locking dynamic compression plate.

### Sensors

Three‐dimensional (3D) motion of the pelvis was recorded using an optical tracking system comprising four cameras (Prime 13®, OptiTrack). This system detects reflective markers at a resolution of 1280 × 1024 pixels, with a manufacturer‐stated accuracy of 0.2 mm. A total of six optical markers were fixed to each hemipelvis using a standardized template, targeting the anterior superior iliac spine, ischial tuberosity, superior pubic ramus, inferior pubic ramus and both sacroiliac joints. Motion data were processed using the system‐specific software (Motive 2.1®, OptiTrack), which reconstructed a corresponding 3D coordinate system. Relative motion of the main pelvic fragments was calculated mathematically in all three spatial dimensions. Maximum displacement was defined as the greatest interfragmentary distance under loading conditions.

As demonstrated in previous studies [24], compressive forces and contact area within the pubic symphysis were dynamically measured using a thin electro‐resistive sensor film, with data acquisition and analysis performed via I‐Scan® software (Tekscan Inc.). To ensure high measurement accuracy and reproducibility, a new sensor was used for each experimental series to minimize potential degradation effects [7, 24, 25].

The sensor films employed were Tekscan type 5033, featuring a matrix height of 38.4 mm and a width of 26.4 mm, with a sensel density of 144.1 sensels/cm^2^, as specified by the manufacturer. Prior to testing, all sensors were conditioned according to manufacturer guidelines. This involved five preconditioning cycles at 120% of the expected maximum load (i.e., 400 N), using rubber‐covered aluminium plates in a universal testing machine (Z 020®, Zwick), to stabilize sensor performance and prevent data loss.

Subsequently, a software‐assisted two‐point calibration was carried out by applying loads of 50 and 500 N. Calibration was performed using symphysis phantoms, which consisted of Synbone® pubic components embedded in Technovit 3040® resin (Heraeus Kulzer), as previously described.

### Segmental pressure mapping

Using the I‐Scan® software, the contact area within the pubic symphysis was divided into three equal segments, corresponding to the cranial, intermediate and caudal thirds of the sensor film. These segments were designated as Region 1 (cranial), Region 2 (intermediate) and Region 3 (caudal). This segmentation enabled subsequent analysis of regional differences in contact pressure distribution within the symphyseal gap, as previously described.

### Experimental setup

In this study, two groups were investigated, each comprising *n* = 5 human anatomical pelvises.

#### Symphyseal locking dynamic compression plate (SLDCP) group

This group was treated using a six‐hole, 3.5 mm SLDCP (Synthes). The plate allowed axial compression via two cortical screws and provided angular stability through four additional locking screws.

#### IF group

In the experimental group, an IF system typically used for the treatment of spinal fractures (USS Fracture MIS; Synthes) was employed as a novel technique for symphyseal stabilization. This approach has been previously described by our working group [11].

We hypothesized that the conventional 3.5 mm symphyseal locking plate would demonstrate inferior biomechanical stability—defined in terms of contact area, compressive force and interfragmentary motion—compared to the IF.

All specimens were prepared as previously described. Sensor films were inserted into the symphyseal gap and temporarily secured using adhesive tape. The gap was then anatomically reduced using reduction tongs (Synthes) applied at predefined positions on either side of the pubic symphysis, until a target compression force of 50 N was achieved. At each step, the resulting compressive forces and contact area within the symphysis were recorded using the I‐Scan® sensor system.

### Fixation using a 3.5 mm SLDCP (SLDCP group)

Step I: The SLDCP was positioned across the pubic symphysis, and both medial cortical screws were inserted to achieve dynamic compression.

**Figure 1:**
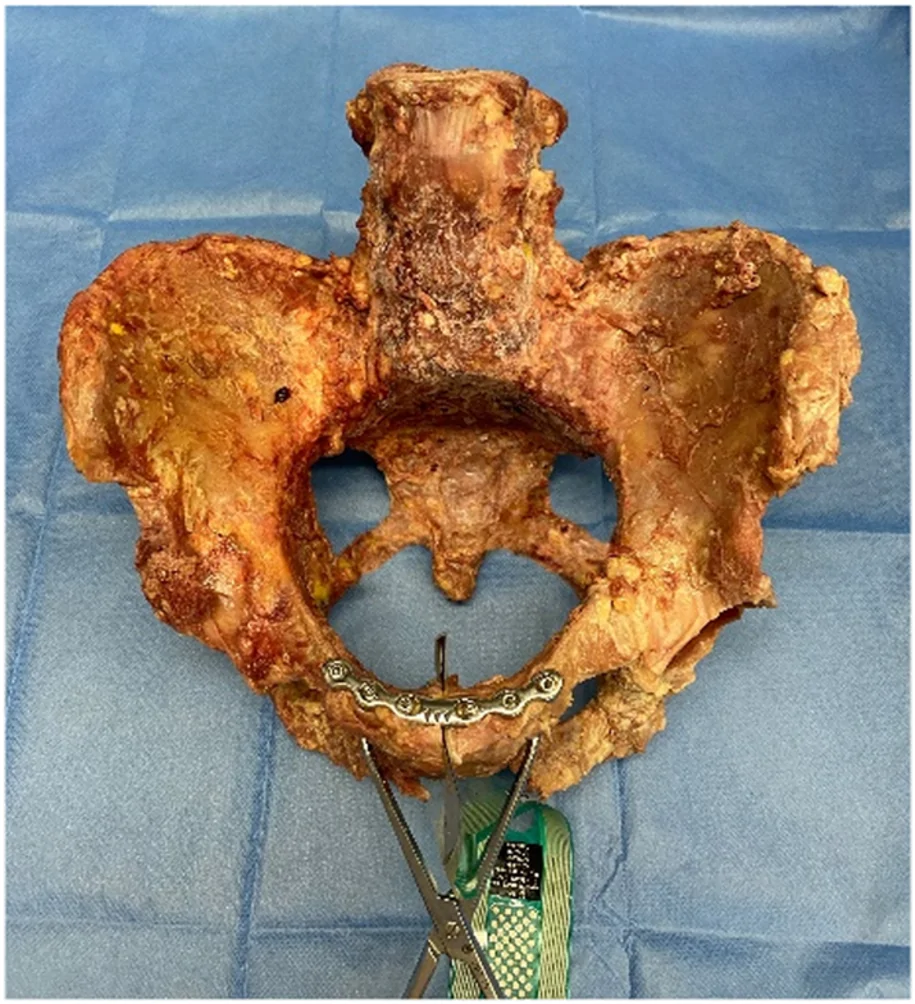
Reduction using a pointed forceps and fixation with a 3.5 SLDCP. SLDCP, symphyseal locking dynamic compression plate.

Step II: Two additional locking screws were placed on each side of the symphysis. For the placement of the interlocking screws, a torque‐controlled screwdriver was used (PSR 960, 3 N m; Bosch) to ensure standardized insertion torque. Upon completion of the osteosynthesis, the reduction tong was removed (Figure 1).

### Fixation using the IF (IF group)

**Figure 2:**
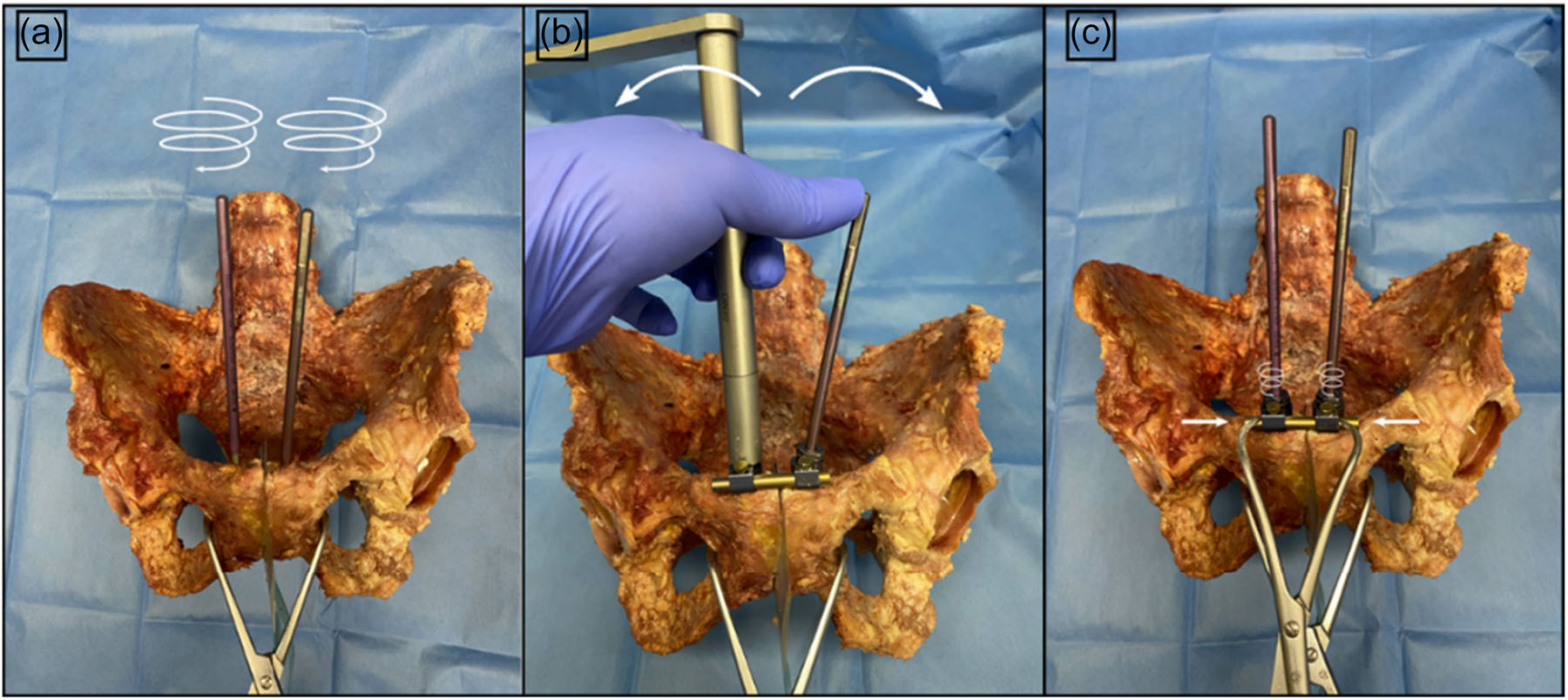
Reduction using a pointed forceps and (a) insertion of Schanz screws on either side of the symphysis, (b) creating caudal compression using socket wrenches and (c) cranial compression.

Initially, a 5 mm transpedicular Schanz screw (Synthes) was inserted on each side of the pelvis in a cranio‐caudal orientation, parallel to the pubic symphysis (Figure 2a). Two fracture clamps were then advanced along the Schanz screws and positioned close to the bone surface. A 6 mm (diameter) titanium fixation rod measuring 35 mm in length (Synthesy) was subsequently placed between the two clamps to establish the construct, followed by the stabilization sequence described below.

Step I: An 11 mm socket wrench was positioned over both Schanz screws and tilted laterally. The clamps and Schanz screws were locked in this orientation, producing caudal compression across the pubic symphysis (Figure 2b).

Step II: To generate cranial compression, both fracture clamps were shifted medially along the vertical rods, and a 6 mm socket wrench was used to secure them in position (Figure 2c). Following this, the reduction tong was removed.

All subsequent steps were performed analogously to those in the other groups.

This 3D reduction technique facilitates the repositioning of displaced fragments and enables controlled caudal compression of the symphysis. Furthermore, by cranially pulling on the secured fracture clamps, additional contact area within the symphyseal gap can be achieved. Analogous to its application in spinal surgery, this technique can be implemented clinically using a minimally invasive approach.

All pelvises were tested using a simulated bilateral stance model, as described in previous studies [5, 11, 12]. Axial loading was applied via a ceramic femoral head prosthesis (28 mm, Zimmer Inc.), articulating at an angle of 45° to the proximal sacrum within a custom‐made cement cup (Technovit 3040®, Heraeus Kulzer GmbH) fitted to the first sacral vertebra.

Each pelvis was positioned with an initial preload of 50 N. The orientation was set to 25° inclination and 15° abduction, representing the physiological loading vector during gait shortly before toe‐off. The defined maximum load and subsequent unloading (relief plateau) were each maintained for 10 s. The first two loading cycles were designated as setting cycles. During the subsequent five cycles, displacement and motion were measured.

Loading began at 50 N and was incrementally increased in 50 N steps up to a maximum of 600 N. This maximum load was derived based on an estimated mean body weight of 80 kg, assuming 20 kg per lower limb. The remaining 40 kg was considered to load the pelvis directly, corresponding to approximately 400 N.

After the final loading cycle, all specimens were removed from the universal testing machine.

### Data acquisition, processing and statistical analysis

Following each reduction and at every subsequent step, compressive forces and contact area within the pubic symphysis were measured using I‐Scan® sensor films (Type 5033, TekScan Inc.). Additionally, regional contact distribution across the cranial, central and caudal thirds of the symphysis was quantified using I‐Scan® software version 6.02 (TekScan Inc.), expressed as the percentage of contact area to serve as an indicator of reduction quality.

Pelvic spatial instability and fragment motion were recorded using an optical tracking system (Prime 13®, OptiTrack). Optical markers were affixed to standardized anatomical landmarks on each pelvis using a positioning template. Data analysis was conducted using the manufacturer's software (Motive 2.1®, OptiTrack).

Statistical analyses were performed with SigmaPlot 13.0 (Systat Software). Descriptive statistics included mean values, ranges and interquartile ranges (25th–75th percentile). To assess differences between stabilization stages within each group, repeated‐measures analysis of variance (ANOVA) with Dunnett's post hoc test was applied, with the post‐stabilization condition defined as the control [2]. Comparisons between groups were conducted using Kruskal–Wallis one‐way ANOVA on ranks, followed by Student–Newman–Keuls post hoc testing. A *p* value < 0.05 was considered statistically significant [2].

## RESULTS

### Bone density and symphyseal area

**Figure 3:**
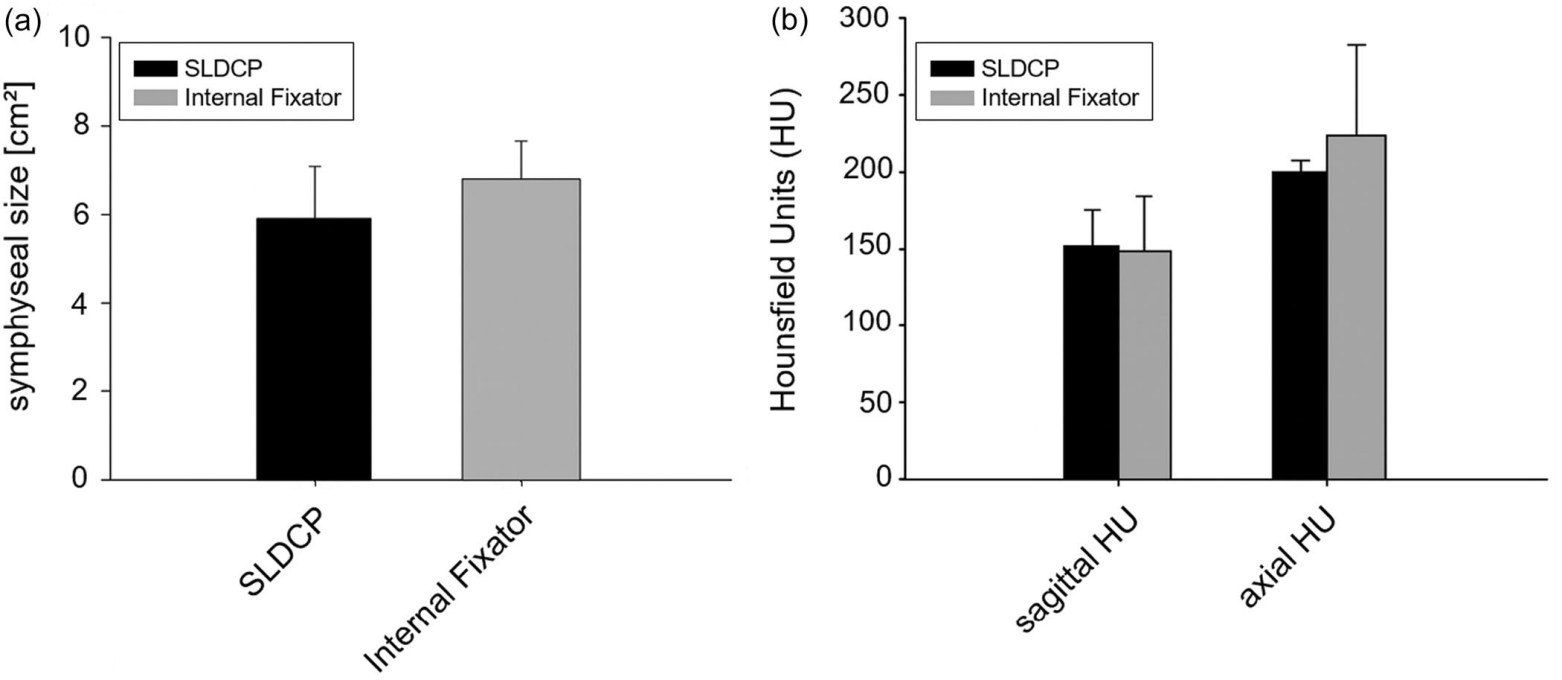
(a) Mean of the pubic symphysis size in the sagittal plane of the CT analysis. (b) Mean of the HU at S1 in sagittal and axial CT analysis. CT, computed tomography; HU, Hounsfield units; SLDCP, symphyseal locking dynamic compression plate.

There was no statistically significant difference regarding the mean symphyseal area or the mean bone density between both groups (Figure 3).

### Intrasymphyseal compression forces and contact area

**Figure 4:**
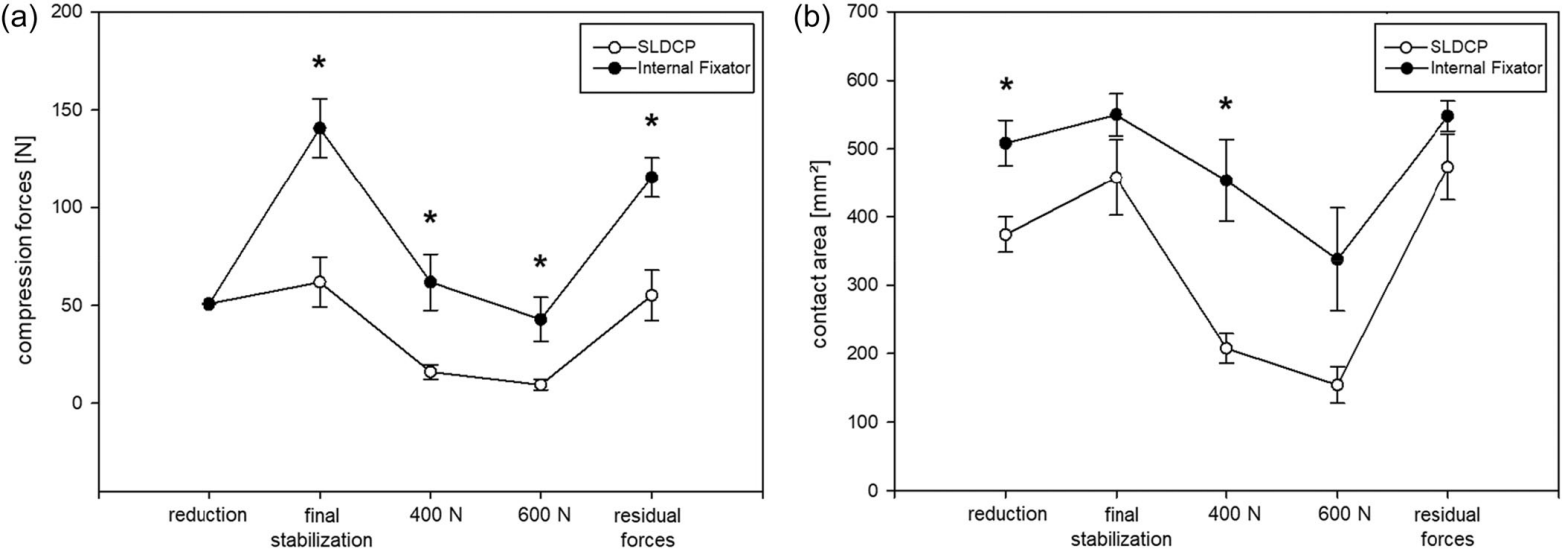
Intrasymphyseal compression forces (a) and contact area (b) (mean ± SEM). *Asterix mark statistical significance. SEM, standard error of the mean; SLDCP, symphyseal locking dynamic compression plate.

With the symphysis reduced, there was no significant difference in compression forces within or between the two groups. Regarding the symphyseal contact area, significantly (*p* = 0.013) higher values could be seen in the IF group (508.2 ± 74.72 mm^2^) as compared to the SLDCP group (373.8 ± 57.7 mm^2^) (Figure 4).

After stabilization with either the IF or the 3.5 SLDCP, the IF group showed significantly (*p* = 0.004) higher compression forces (140.72 ± 33.47 N) compared to the SLDCP group (61.82 ± 28.41 N). In contrast, there was no statistical difference (*p* = 0.183) regarding the symphyseal contact area between the IF group (550.0 ± 68.47 mm^2^) and the SLDCP group (457.6 ± 123.9 mm²).

Reaching the 400 N axial loading forces plateau, the compression forces measured in the IF group (61.78 ± 31.98 N) were higher than in the SLDCP group (15.98 ± 8.2 N). The difference was statistically significant (*p* = 0.015). In addition, the measured symphyseal contact area was significantly (*p* = 0.008) higher in the IF group (453.0 ± 137.6 mm^2^) compared to the SLDCP group (216.0 ± 135.0 mm^2^).

After reaching 600 N axial loading during the testing phase, the forces measured in the IF group (48.82 ± 25.2 N) were higher than in the SLDCP group (9.42 ± 5.81 N), which was statistically significant (p = 0.02). The symphyseal contact area showed no statistically significant (*p* = 0.051) difference between both groups (IF group 337.6 ± 168.45 mm^2^ vs. SLDCP group 154.0 ± 59.73 mm^2^).

At the end of the testing phase with no more loading applied, the forces measured within the IF group remained significantly higher (*p* = 0.006) compared to the SLDCP group (115.44 ± 22.82 N vs. 55.09 ± 28.81 N). In contrast, there was no statistically significant (*p* = 0.196) difference between the IF group (548.0 ± 49.05 mm^2^) and the SLDCP group (473.2 ± 108.08 mm^2^) regarding the symphyseal contact area.

### Segmental compression forces

#### Cranial segment

**Figure 5:**
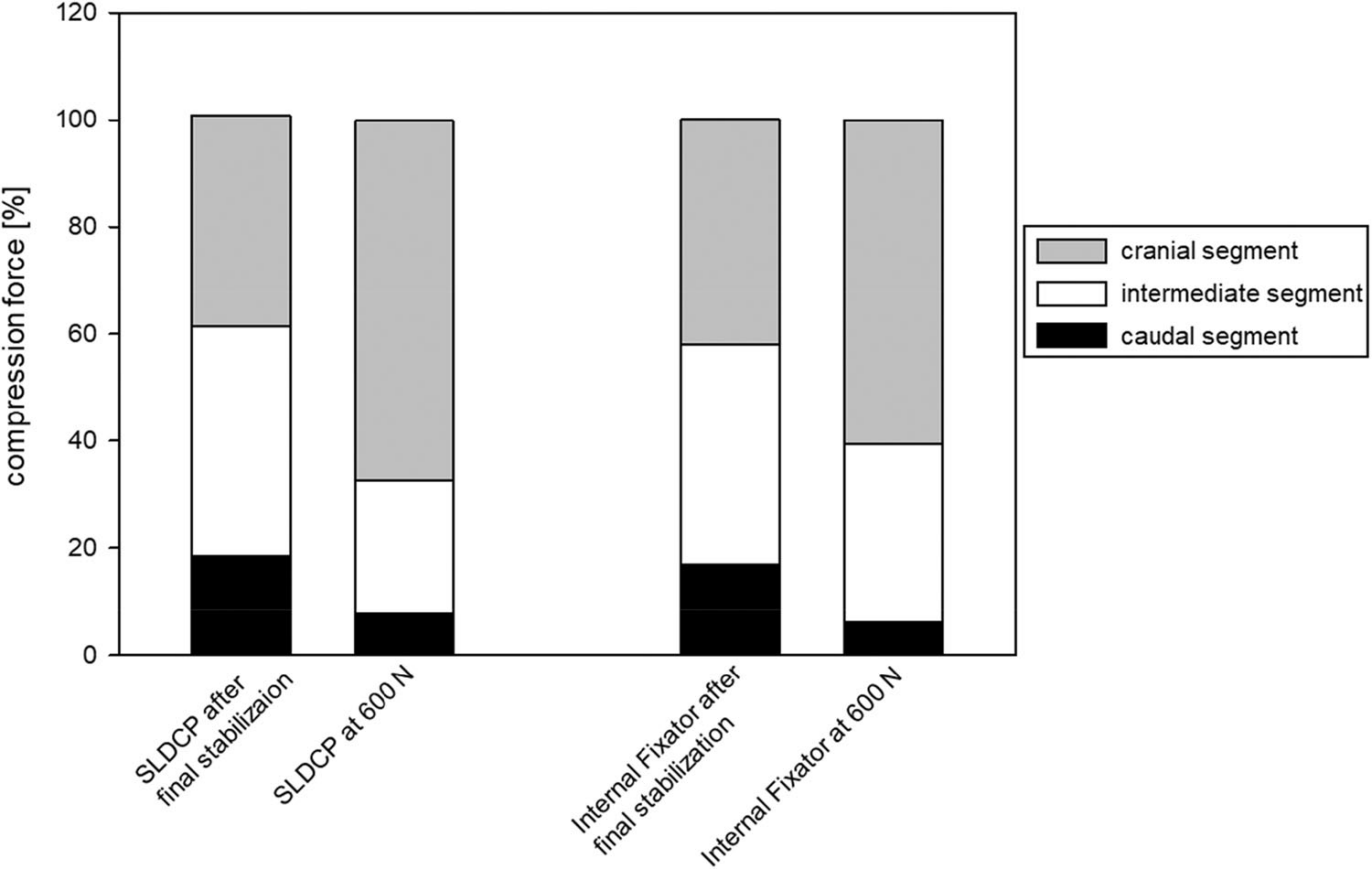
Segmental compression force distribution (mean ± SEM). SEM, standard error of the mean; SLDCP, symphyseal locking dynamic compression plate.

With no axial loading applied, compression forces in the IF group (42% of the total load measured in the symphyseal gap) were higher than in the SLDCP group (39%). During loading, reaching the maximum of 600 N, forces changed to 61% in the IF group and 68% in the SLDCP group (Figure 5). There was no statistical significance (*p* > 0.05).

#### Intermediate segment

In the IF group, values changed from 41% of total load measured in the symphyseal gap before loading to 33% under maximum loading and in the SLDCP group from 43% to 25%, both without significance between the two groups and to final stabilization (*p* > 0.05).

#### Caudal segment

The symphyseal compression forces measured in the IF group changed from 17% before loading to 6% at full loading forces. In the SLDCP group, a decline from 18% to 8% under maximum loading was observed. There was no statistical significance (*p* > 0.05).

### Segmental contact area

#### Cranial segment

**Figure 6:**
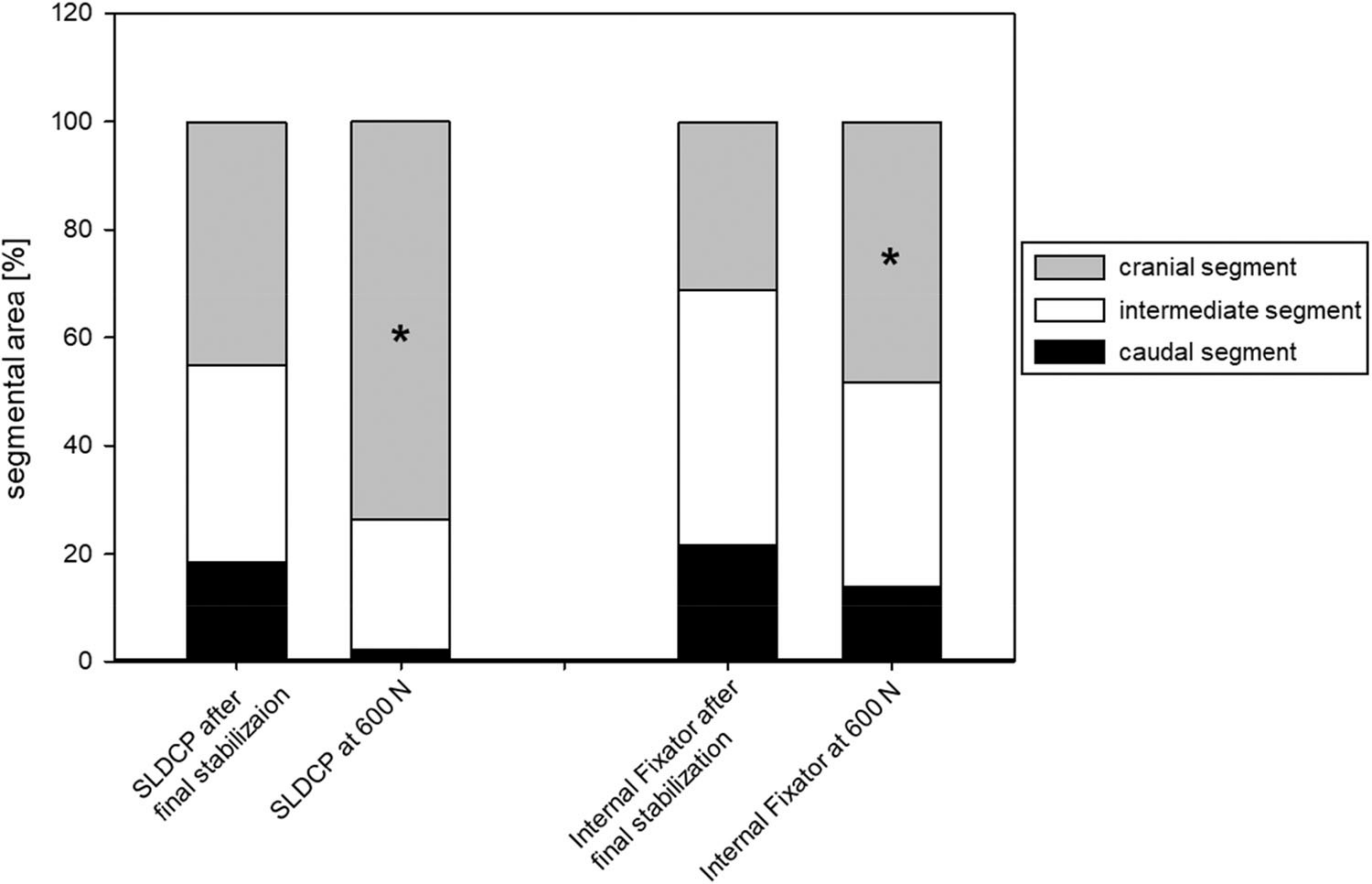
Segmental area distribution (mean ± SEM). SEM, standard error of the mean; SLDCP, symphyseal locking dynamic compression plate. *p < 0.05 versus final stabilization.

Before loading, the symphyseal contact area in the cranial segment in the IF group was 31% (of total contact area) and 45% in the SLDCP group. With increasing force applied, it reached 49% (600 N) in the IF group and 73% (600 N) in the SLDCP group (Figure 6). In both the SLDCP and IF groups, this marked a significantly higher contact area in the cranial segment at 600 N compared to the starting point after final fixation (*p* < 0.05).

#### Intermediate segment

In the intermediate segment, the contact area decreased with increasing force in both groups. In the IF group, it declined from 46% at the start to 38% (600 N). In the SLDCP group, the contact area also decreased from 36% to 25% (600 N). There was no statistical significance (*p* > 0.05).

#### Caudal segment

In the caudal segment, a different dynamic was observed between the two groups, with the IF group showing decreasing contact area from 21% in the beginning to 13% at 600 N. In the SLDCP group, after measuring 18% in the beginning, it declined rapidly to 2% at 600 N, neither of which showed statistical significance (*p* > 0.05).

### 3D motion analysis

Under axial loading, the cranial symphyseal gap was reduced. At 600 N, a further reduction of 0.054 mm (IF group) and 0.142 mm (SLDCP group) was noticed.

The caudal symphyseal gap showed a reverse effect, where a widening of the symphyseal gap at 400 N was observed in the IF group to measure 0.15 mm and in the SLDCP group with 0.27 mm. At 600 N, the gap further increased by 0.27 mm in the IF group and by 0.51 mm in the SLDCP group. The difference between the two groups at 600 N was statistically significant (*p* < 0.05).

In the SI joint, a decreasing width was observed with increasing axial loading by 0.43 mm (IF group) and 0.79 mm (SLDCP group) at 600 N. The difference was not statistically significant (*p* > 0.05).

## DISCUSSION

The findings of this study confirm our hypothesis that reduction and fixation of acute traumatic symphyseal instabilities using an IF provides superior biomechanical stability compared to conventional symphyseal plating. The IF group demonstrated significantly higher compressive forces within the symphyseal gap, a more homogeneous force distribution across the segments, and a greater contact area, particularly in the caudal region, throughout the loading cycle. These biomechanical characteristics are likely to reduce the risk of implant loosening and secondary diastasis, thereby potentially supporting improved healing compared to the standard symphyseal plate osteosynthesis.

Symphyseal plating, despite being widely used as a definitive treatment, is frequently associated with implant failure and complications such as screw migration into the bladder [6, 23]. Although implant failure does not necessarily correlate with worse outcomes [10, 21], early mechanical failure can result in diastasis and the need for revision surgery [9]. By contrast, the IF construct in our study provided greater initial stability, which may translate into a lower risk of such complications.

Previous studies have investigated alternative fixation strategies. The INFIX system, for example, has demonstrated complication rates similar to those of plating, including deep infections, hardware problems and heterotopic ossifications [30]. Other approaches, such as trans‐obturator cable fixation [16] or percutaneous screw osteosynthesis [27], offer promising results but require open exposure or are technically demanding. Compared with these techniques, the IF combines the minimally invasive benefits of external fixation with enhanced stability by adapting implant systems from spinal surgery. Our findings confirm earlier reports from composite pelvis models [10] and extend them to osteoporotic human bone specimens, which better replicate clinical conditions.

Both higher compressive forces and a more uniform contact distribution may contribute to improved healing [3]. Compression of fracture gaps leads to higher strain and therefore favours direct bone healing, providing rigid constructions. Pizanis et al. [24] demonstrated that prebending conventional symphyseal plates increases contact and compression forces—a principle also known to enhance healing in long bone fracture models, especially in transverse fractures.

An important strength of this study lies in the use of cadaveric pelvises rather than synthetic models. While synthetic bone models provide standardization, they lack the variability and ligamentous structures present in human tissue [13, 24]. By employing CT‐based HU analysis, we ensured comparability of bone quality and symphyseal contact area across groups. Although donor specimens typically exhibit reduced bone quality compared to younger trauma patients, this may actually underestimate the biomechanical stability achievable in vivo. Furthermore, cadaveric pelvises are heterogeneous compared to synthetic pelvises. However, with existing sacroiliac ligaments, higher axial loading can be applied to the pelvises, compared to synthetic pelvises.

Regarding symphyseal contact area as an indicator of reduction quality and healing potential, as it has already been evaluated for healing capabilities in long bone models [14, 28], higher contact was initially observed in the IF group following reduction, reflecting the effectiveness of the two‐stage reduction manoeuvre. After final fixation, the contact area remained higher in the IF group throughout the testing cycle, though statistical significance was only reached at 400 N. However, as already mentioned, this mark was shown to be a relevant load threshold for symphyseal fixation [13].

Analyzing the contact area distribution across the three predefined symphyseal segments, the IF group showed a more homogeneous distribution. Notably, the caudal segment exhibited minimal contact in the SLDCP group at 600 N, while the IF group retained greater caudal contact.

Our bilateral stance model [5, 11, 12], although limited in replicating combined vertical and horizontal pelvic loads, is well suited for analyzing horizontal stability and anterior pelvic ring motion. The observed superiority of the IF construct under these conditions highlights its potential to withstand clinically relevant forces up to 400 N [17], which are known thresholds for symphyseal fixation.

A limitation of the present study is the restricted loading protocol, which does not account for rotational forces. Additionally, the use of human cadaveric specimens introduces heterogeneity in bone quality and morphological dimensions. To mitigate this issue, we allocated pelvises to both groups based on bone mineral density and symphyseal dimensions.

Retrospectively, the results of our post‐hoc power analysis suggest that a larger sample size would have been necessary to achieve a statistical power of 0.8. Specifically, a total of *n* = 28 cadaveric pelvises would have been required. However, considering responsible resource utilization in experimental research, such a sample size would be impractically large and therefore unjustified.

In summary, the IF provides superior compressive forces and a more uniform contact distribution across the symphysis compared to plating. These features may translate into improved implant stability and healing in clinical practice. Further clinical studies are needed to validate these biomechanical advantages and assess long‐term outcomes.

## AUTHOR CONTRIBUTIONS

*Conceptualization*: Tobias Fritz and Antonius Pizanis. *Methodology*: Tobias Fritz. *Data acquisition*: Jonas Stroeder. *Formal analysis*: Tobias Fritz and Jeremy Briem. *Investigation*: Tobias Fritz and Jeremy Briem. *Data curation*: Tobias Fritz and Jeremy Briem. *Writing—original draft preparation*: David B. Osche. *Writing—review and editing*: David B. Osche, Tobias Fritz, Emmanouil Liodakis, Laura Mettelsiefen, Alexa J. Fischer, Marcel Orth, Tim Pohlemann and Antonius Pizanis. *Supervision*: Tobias Fritz. *Project administration*: Tobias Fritz. All authors have read and agreed to the published version of the manuscript.

## CONFLICT OF INTEREST STATEMENT

The authors declare no conflicts of interest.

## ETHICS STATEMENT

The cadavers utilized in this study were from body donors who had provided written informed consent during their lifetime for postmortem use in research and education. They were obtained through the body donation programme at Saarland University. This study was approved by the local ethics committee (vote number 131/21; ‘Ständige Ethikkommission der Ärztekammer des Saarlandes’, date of approval 22 April 2021) and conducted in accordance with the guidelines of the Declaration of Helsinki.

## Data Availability

The data sets generated and analyzed during the current study are available from the corresponding authors upon reasonable request.
